# Efficient Production of Fluorescent Transgenic Rats using the *piggyBac* Transposon

**DOI:** 10.1038/srep33225

**Published:** 2016-09-14

**Authors:** Tianda Li, Ling Shuai, Junjie Mao, Xuepeng Wang, Mei Wang, Xinxin Zhang, Leyun Wang, Yanni Li, Wei Li, Qi Zhou

**Affiliations:** 1 State Key Laboratory of Reproductive Biology, Institute of Zoology, Chinese Academy of Sciences, Beijing 100101, China; 2 State Key Laboratory of Medicinal Chemical Biology, Nankai University, Tianjin 300350, China; 3 Graduate School of Chinese Academy of Sciences, Beijing 100049, China; 4 College of Life Science, Northeast Agricultural University of China, Harbin 150030, China

## Abstract

Rats with fluorescent markers are of great value for studies that trace lineage-specific development, particularly those assessing the differentiation potential of embryonic stem cells (ESCs). The *piggyBac* (*PB*) transposon is widely used for the efficient introduction of genetic modifications into genomes, and has already been successfully used to produce transgenic mice and rats. Here, we generated transgenic rats carrying either the *desRed* fluorescent protein (*RFP*) gene or the enhanced green fluorescent protein (e*GFP*) gene by injecting pronuclei with *PB* plasmids. We showed that the transgenic rats expressed the *RFP* or e*GFP* gene in many organs and had the capability to transmit the marker gene to the next generation through germline integration. In addition, rat embryonic stem cells (ESCs) carrying an *RFP* reporter gene can be derived from the blastocysts of the transgenic rats. Moreover, the *RFP* gene can be detected in chimeras derived from *RFP* ESCs via blastocyst injection. This work suggests that *PB*-mediated transgenesis is a powerful tool to generate transgenic rats expressing fluorescent proteins with high efficiency, and this technique can be used to derive rat ESCs expressing a reporter protein.

*PiggyBac* (*PB*) is a DNA transposon element that was first isolated from *Trichoplusia ni*^1^,^2^ and has shown an efficient transposition ability in many species^3^,^4^,^5^. Compared with other transposons (e.g., *sleeping beauty*, *Tol2* and *MosI*), *PB* can insert with higher transposition activity into mammalian genomes^6^, which makes it an ideal tool for research on the genetic traits of mammalian cells^7^,^8^,^9^. Therefore, *PB* has been extensively utilized in studies of genetic function and pathway regulation, such as the reprogramming of somatic cells into pluripotent stem cells^10^, the discovery of novel regulators that govern the pluripotency of stem cells^11^ and the generation of a mutation library for genetic screening^12^.

The rat is an important and useful laboratory species for modeling clinical diseases^13^. Although the rat genome has been sequenced (Rat Genome Sequencing Project Consortium 2004) and shared (www.ensembl.org/Rattus_norvegicus), little work has been performed to predict genetic functions compared with the mouse. To date, several gene manipulation strategies (such as *N*-ethyl-*N*-nitrosourea (ENU)^14^, zinc-finger nucleases (ZFNs)^15^ and transcription activator-like (TAL) effector nucleases (TALENs)^16^) have been used to produce genetically modified rats. Meanwhile, embryo manipulation techniques, including traditional pronuclear injection^15^, ESC germline transmission^17^ and haploid ESC intracytoplasmic injection^18^, have been developed for the production of transgenic rats. However, these methods are difficult to perform and are time-consuming. To date, compared with the thousands of types of transgenic mice, rats carrying fluorescent markers are still lacking in biological research, thus hindering the study of the developmental potential of rats during embryogenesis and differentiation.

Here, we have developed a simple and efficient strategy to generate transgenic rats that carry fluorescent marker genes. We microinjected a modified *PB* vector carrying either the *desRed* fluorescent protein (*RFP*) gene or the enhanced green fluorescent protein (e*GFP*) gene with a *PBase* plasmid into the pronuclei of rat zygotes. The *RFP* and *eGFP* genes were able to insert into many sites of the rat genome and were expressed in various organs. This stable insertion by the *PB* system was passed to the next generation through germline transmission. In summary, this study provided a novel method to produce fluorescent rats that can be used as a fluorescent ESC platform to target differentiation potential.

## Results

### Generation of RFP and eGFP rats with the PB transposon

**Figure 1:**
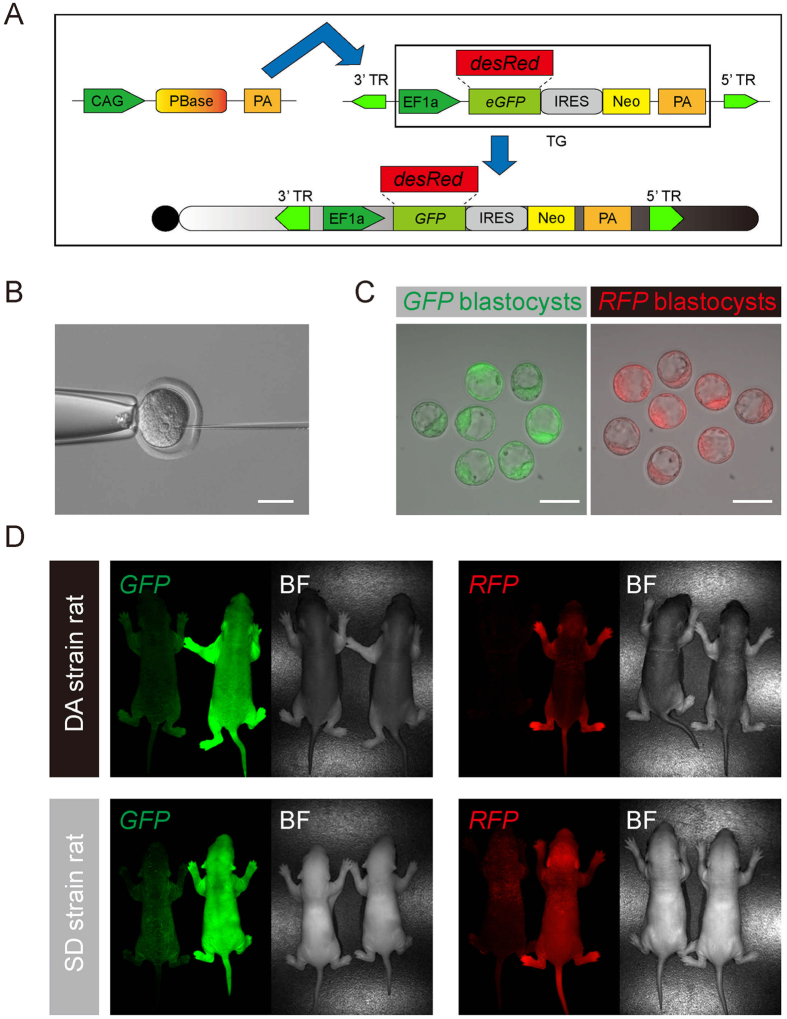
Derivation of *GFP*- and *RFP*-labeled rats via the *piggyBac* transposon. (**A**) Diagram of the *PB* vectors and *PBase* vector. The *PB* vector carried either a *GFP* or an *RFP* transgene driven by an EF1α promoter and efficiently transported the exogenous genes into the chromosome. (**B**) Microinjection of the *PB* and *PBase* vectors into the pronuclei of male zygotes; scale bar = 50 μm. (**C**) Images of transgenic embryos developing into blastocysts with strong *GFP* or *RFP* expression; scale bar = 100 μm. (**D**) Images of *GFP* and *RFP* rat pups on the DA and SD backgrounds that were generated by *PB* transposition.

As a fluorescent marker, the *RFP* gene has been frequently used in cell and molecular biology^19^. We designed a system to insert a *PB* vector containing the *RFP* or e*GFP* gene into rat genomes using the traditional pronuclear injection method. As shown in Fig. 1A, donor transgene (which was abbreviated TG) DNA (*RFP* or *eGFP*) was inserted into the *PB* vector adjacent to the *EF1α* promoter (Fig. 1A). For each injection, 30 ng/μl *PB* plasmid and 10 ng/μl *PBase* plasmid were co-injected into the pronuclei of zygotes to test the efficiency of the *PB* integration (Fig. 1B). The zygotes were obtained from Sprague Dawley (SD) and Dark Agouti (DA) strain rats. Two hundred twenty-three zygotes were manipulated via plasmid injection. One hundred ninety-seven embryos survived (88.3%) and developed to the blastocyst stage (Fig. 1C, Table 1). All blastocysts were transferred back to the uteri of pseudo-pregnant rats, and 98 of them developed to term. Of the generated pups, 44 founder pups (F0) carried the fluorescent markers, among which 26 were SD strain rats (white coat) and 18 were DA strain rats (agouti coat) (Table 1). These pups showed strong red or green fluorescence, as detected by a stereo fluorescence microscope (S165, Leica, Germany). The emission wavelength peaks were detected between 510 nm and 600 nm using *in vivo* imaging instruments (IVIS, PerkinElmer, USA), indicating that the marker genes had been integrated into the genomes by transposition (Fig. 1D, supplementary Fig. S1A,S1B).

**Figure 2:**
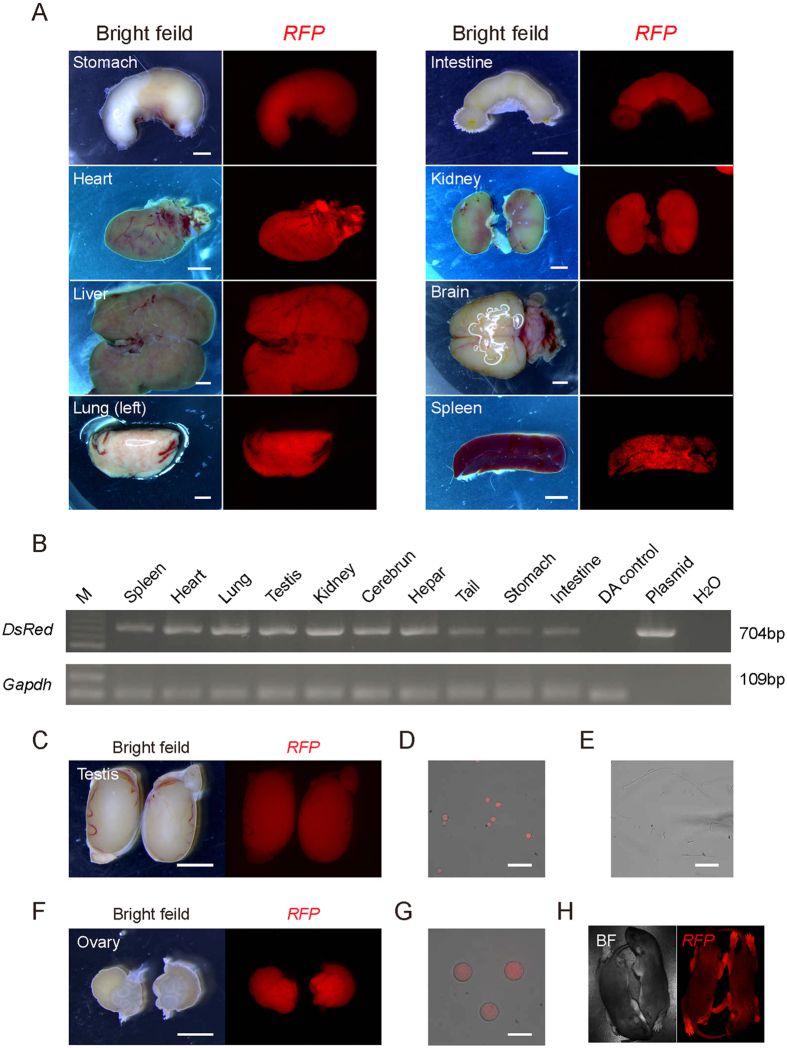
Integration of the marker genes and germline transmission. (**A**) Images of the organs (stomach, heart, liver, lung, intestine, kidney, brain and spleen) dissected from one *RFP* transgenic rat (*RFP*-positive offspring from founder rats); scale bar = 1 mm. (**B**) PCR analysis of the integration of the *desRed* gene in different organs shown in panel A. All tested organs from the RFP rats were *desRed* gene positive compared with the wild type Dark Agouti control (the lane which was marked by DA control). (**C**) Images of the testes from the *RFP* founder rat; scale bar = 1 mm. (**D**) Image of RFP-positive round sperm from the testes of the *RFP* founder rat; scale bar = 100 μm. (**E**) Images of normal mature sperm that had separated from the *RFP* founder rat testes; scale bar = 100 μm. (**F**) Images of the ovaries from the *RFP* founder rat; scale bar = 1 mm. (**G**) Image of RFP-positive oocytes at the germ-vesicle (GV) stage from the ovaries of the *RFP* founder rat; scale bar = 100 μm. (**H**) F1 *RFP* pups generated by mating *RFP* rats with WT rats.

We sacrificed one *RFP* rat to assess whether the *PB* transposon had integrated into different organs. All eight organs assessed (the stomach, heart, liver, lung, intestine, kidney, brain and spleen) showed strong red fluorescence (Fig. 2A), indicating that the *PB* transposon had been introduced efficiently. A PCR analysis of the *RFP* gene in the eight organs further confirmed this result (Fig. 2B). These data showed that *RFP* and *eGFP* (see supplementary Fig. S2A) rats can be efficiently generated using genomic transposition.

### Germline transmission of RFP and eGFP

We assessed the gametes of the founder rats to determine whether the *RFP* and *eGFP* marker genes can be transmitted to the progeny through the germline. The data revealed that testes and germ cells from male rats were *RFP*-positive under a fluorescence microscope (Fig. 2C,D). However, mature spermatids without a cytoplasm were *RFP*-negative, indicating that *RFP* was located inside the cytoplasm (Fig. 2E). Furthermore, we performed a fluorescence-activated cell sorting (FACS) analysis to determine the percentage of *RFP*-positive cells in haploid germ cells (round spermatids and mature spermatids). In the *RFP* testes group, 34.4% of the haploid peak gated cells were *RFP*-positive, whereas no *RFP*-positive cells were observed in the wild type (WT) control haploid cells (see supplementary Fig. S2B). These data showed that male *RFP* rats can generate gametes with the *RFP* modification. In a parallel experiment, female *RFP* rats were assessed for the formation of *RFP* germ cells. The data showed that ovaries and germ cells at the mature MII oocyte and germinal vesicle (GV) stages (Fig. 2F,G and supplementary Fig. S2C) were *RFP*-positive, which implied that female *RFP* rats can also form gametes with the *RFP* modification by *PB* transposon. Furthermore, the *RFP*-positive rats grew to adulthood, and full-term pups (F1) carrying *RFP* expression were obtained after crosses with WT rats (Fig. 2H and supplementary Table S1). In this assay, the fluorescent *eGFP* gene is also an ideal genetic marker for rats (data not shown). In conclusion, the *PB*-introduced *RFP* or *eGFP* marker gene can be stably inherited by the next generation through germline transmission.

### Integration of the PB transposon in the rat genome

**Figure 3:**
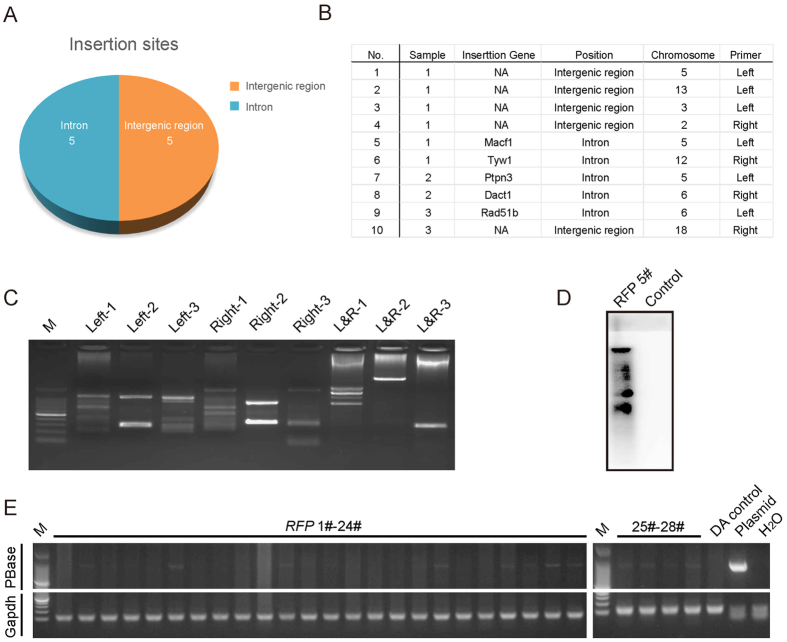
Analysis of *PB* insertions in the generated pups. (**A**) Summary of the *PB* transposon integration sites. Five rats exhibited an insertion in an intron region and another five exhibited an insertion in an intergenic region. (**B**) Exact insertion sites of ten independent rats. (**C**) Inverse PCR analysis of the transposition sites in the transgenic rats. (**D**) Detection of the number of *PB* transposon copies in an RFP-positive offspring. (**E**) Exploration of the integration of *PBase* in the transgenic rats; each band indicated the random integration of the *PBase* gene.

We investigated the insertion sites of the *PB* transposon in the genomes of the *RFP* and *eGFP* rats. Among the 10 insertion sites, 5 were inserted into an intron and 5 were inserted into intergenic sites (Fig. 3A). The five genes with *PB* insertion in an intron were Macf1, Tyw1, Ptpn3, Dact1 and Rad51b, which are located on chromosomes 5, 12, 5, 6 and 6, respectively (Fig. 3B). Some of the rats had a single copy insertion, but many others had multiple copy insertions (Fig. 3C). We explored the genomes of 21 *RFP* rats and 24 *eGFP* rats by inverse PCR to evaluate the efficiency of the transgene transfer (see supplementary Fig. S3A). Our data showed that the *PB* transposon could efficiently deliver the exogenous genes into the rat genome (see supplementary Fig. S3B,S3C). A southern blot assay was performed to identify the number of *PB* transposon copies. Our data showed that the *PB* transposon could integrate into the genome at multiple sites (Fig. 3D), which is consistent with the inverse PCR result (see supplementary Fig. S3B). Furthermore, we explored the entire genome to test whether the *PBase* vector integrated. Although *PBase* integration occurred in both *RFP*- and *eGFP*-positive rats, some of the offspring were *PBase*-free, with stable integration of the marker genes (Fig. 3E and supplementary Fig. S3D).

### RFP-positive ESCs derived from the PB-integrated rats

**Figure 4:**
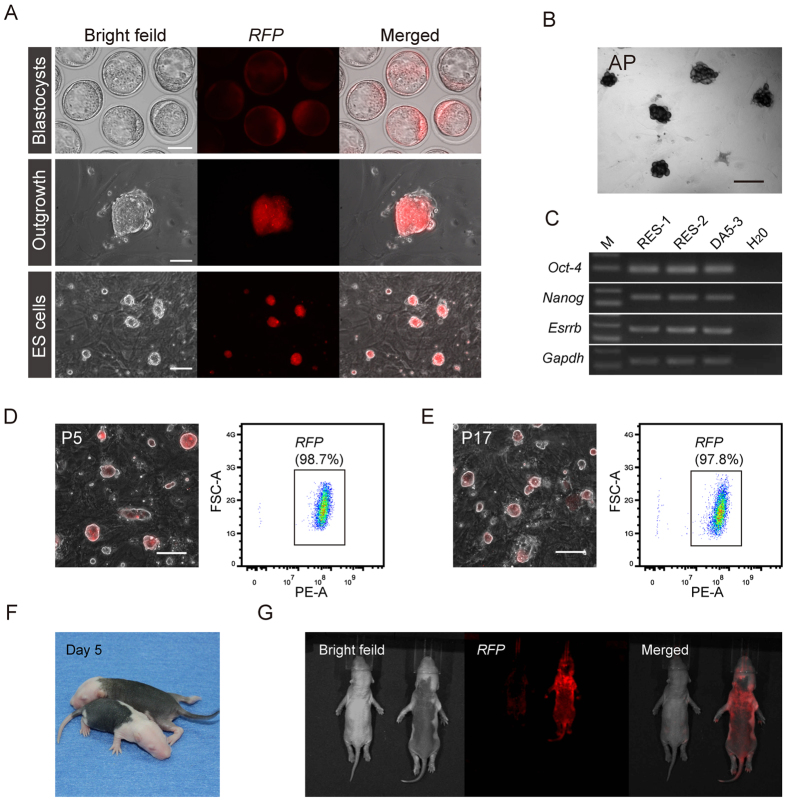
Derivation of ESCs from the *RFP* rats. (**A**) RFP-positive ESCs derived from E4.5 blastocysts of *RFP* rats. Top panel: rat blastocysts; middle panel: outgrowth (day 5); and bottom panel: established rat ESCs; scale bar = 50 μm. (**B**) Alkaline phosphatase (AP) staining of RFP-rat ESC colonies; scale bar = 50 μm. (**C**) RT-PCR analysis of pluripotent marker gene expression in the derived RFP-ESCs. (**D**) Percentages of RFP-positive cells (PBES1-1) at the initial passage (P5, 98.7%) were detected by FACS analysis; scale bar = 50 μm. (**E**) Percentages of RFP-positive cells in PBES1-1 cells after 12 passages (P17, 97.8%); scale bar = 50 μm. (**F**) Chimeric rat pups derived from the RFP-rat ESCs. The chimeric rats showed a high contribution of RFP-rat ESCs, according to the coat color. (**G**) Expression of the *desRed* gene in the chimeric pups. The chimeric pups showed a high level of *RFP* expression compared with the negative control.

We next performed rat ESC derivation experiments using *RFP* blastocysts harvested from the *PB*-integrated DA strain rats. *RFP* was stably expressed in both E4.5 blastocysts and derivative outgrowths after 5 days of culture in N2B27 medium supplemented with “2i” (PD0325901 and CHIR99021) and leukemia inhibitory factor (LIF)^20^,^21^ (Fig. 4A). Standard rat ESC cell lines that expressed *RFP* were established and passaged *in vitro* for long periods. The expression of alkaline phosphatase (Fig. 4B) and pluripotent marker genes (Fig. 4C) indicated that the *PB* insertion did not affect the core pathways regulating pluripotency. We assessed whether *RFP* would be silenced during the ESCs culture procedure using FACS analysis. The results indicated that the percentage of *RFP*-positive cells at passage five was 98.7% (Fig. 4D) and was 97.8% after passage 12, with no subsequent significant decrease (Fig. 4E). Hence, *PB* integration can stably introduce *RFP* genes into the genome of the derivative ESCs, and the expression remained constant.

We produced chimeras by injecting blastocysts to examine the differentiation potential of these *RFP*-positive rat ESCs (see supplementary Fig. S4A). In two independent donor cell lines, 39 offspring were derived from 86 reconstructed and transferred embryos (Table S2). Ten chimeric rats were generated, with the contributions distinguished by coat color (Fig. 4F) and fluorescence detection (Fig. 4G). In summary, genomically integrated, pluripotent *RFP*-positive ESCs could be derived from *PB*-integrated *RFP* rats. This technique enables the generation of a supply of fluorescently labeled rat ESCs for future research.

In this study, we developed a simple and efficient method to generate transgenic rats that carry fluorescent marker genes. Our results showed that *RFP* and *eGFP* rats can be produced by microinjection of *PB* vectors carrying *RFP* or *eGFP* genes into the pronuclei of zygotes. We further showed that the *PB* system and fluorescence genes can synergistically form a powerful tool for genetic research in mammals.

## Discussion

*Sleeping beauty* was the first DNA-based transposon system used for genomic engineering in mammalian cells. However, because *PB* was proven to efficiently deliver genes in mice in 2005^22^, it has been widely used in gene modification. Without requiring DNA synthesis, the *PB* transposon can excise itself by forming a hairpin structure^2^, which provides a seamless excision with no “footprint”. Recently, the combination of CRISPR/Cas9 and the *PB* transposase system was used to produce a model to track and transform neocortical progenitors and provided a new strategy to study genetic function^23^,^24^. The protocol of co-injecting *PB* and *PBase*-encoding mRNA into pronuclei was specifically developed to improve the efficiency of producing transgenic animals and prevent re-transposition events, and can obtain average transformation frequencies of 80%^25^. Cytoplasmic microinjections of *hyPBase* mRNA and p*PB*-CAG-Tag*RFP* DNA showed that 94.4% of blastocysts were Tag*RFP*-positive^26^. However, it is a leap from the cellular level to an individual animal, and the injection of the *PBase* mRNA has great potential as an easier and highly effective method to generate transgenic animals. In this study, *PBase* did not show active state in the generated *eGFP*/*RFP* transgenic rats, which might relate to powerful reproductive ability of rodents. But application of this technique in larger farm animals warrants more investigations. To date, the *PB* system has proven itself as a versatile genetic tool for various applications, such as mutagenesis and transgenesis. Compared with *sleeping beauty* and other conventional viral vectors, such as retrovirus AAV and adenovirus, the *PB* system has a larger cargo size and a protein domain fusion that can flexibly modify the transposase to achieve site-directed integration^2^.

The *PB* transposon is preferentially biased toward transcription units, particularly in regions that contain genes. We found that out of 10 randomly selected rats, five had insertion sites in introns and five had insertion sites in intergenic regions. This system produced fluorescent rats with no deficiencies. In addition, some rats had a single copy insertion, whereas others had multiple insertions. As *PB* is prone to integrate near or within coding units, we have not clearly determined the reason why the rats did not carry insertion sites in exons or promoter regions.

Our study provided a new method to generate genetically modified rats expressing a fluorescent protein in different organs. *RFP* pups were produced by mating *RFP* rats with WT rats, which indicated that the fluorescent marker genes can be stably inherited by the next generation through germline transmission. This technique will enable the production of a supply of fluorescently labeled rat ESCs for further studies.

## Methods

### Rats

Sprague-Dawley (SD), Fisher 344 (F344) and Dark Agouti (DA) strain rats and CF-1 strain mice were purchased from the Beijing Vital River Laboratory Animal Center. All experiments involving animals were conducted according to the Guidelines for the Care and Use of Laboratory Animals established by the Beijing Association for Laboratory Animal Science and approved under the Animal Ethics Committee of the Institute of Zoology, Chinese Academy of Sciences (1 Beichen West Road, Chaoyang District, Beijing, P. R. China).

### Construction of the vectors

Basic *PB* (PB533-A) and transpose vectors were purchased from SBI System Biosciences and then modified. The *desRed* (*RFP*) or enhanced green fluorescent protein (*eGFP*) genes were inserted into the PB533A-1 vector at the EcoRI and BamHI sites.

### Pronuclear microinjection

The *PB* plasmid (30 ng/μl) carrying the *RFP* or *eGFP* genes and the *PBase* vector (10 ng/μl) were co-injected into pronuclei of zygotes harvested from 0.5 dpc (days post-coitus) SD and DA strain rats to produce transgenic embryos. The reconstructed embryos were transferred to the oviducts of pseudo-pregnant female rats.

### Inverse PCR and genotyping

The schematic of the inverse PCR was illustrated in Fig. S3A. Briefly, the genomic DNA from each sample was extracted using the MicroElute Genomic DNA kit (Omega, USA) and digested with BstYI for 16 hours at 37 °C. The BstYI enzyme was then inactivated at 80 °C for 30 min. The ligation reaction conditions were 4 °C for 16 hours after the direct addition of T4 DNA Ligase (NEB, USA) into the inactivated digestion reaction. The PCR experiments (primers are listed in the supplementary information; see supplementary Table S3.) were performed under the following conditions: 95 °C for 5 min, followed by 25 cycles of 95 °C for 30 sec, 58 °C for 30 sec and 72 °C for 2 min, and 72 °C for 10 min for terminal replication (first round); and 95 °C for 5 min, followed by 32 cycles of 95 °C for 30 sec, 60 °C for 30 sec, and 72 °C for 90 sec, and 72 °C for 10 min for terminal replication (second round). The products of the inverse PCR were cloned into the pMD™18-T vector (TaKaRa, China) and then sequenced by Sanger sequencing with the M13F primer. The sequence files were analyzed and aligned using the BLAST tool (NCBI, USA) on the official website.

The fluorescent cassettes in the transgenic rats were amplified in a PCR assay under the following conditions: 95 °C for 5 min, 32 cycles of 95 °C for 30 sec, 59 °C for 30 sec, and 72 °C for 1 min, followed by 72 °C for 10 min.

A pair of primers (supplementary Table S3) was designed to amplify the *PBase* DNA in the genome, under the following conditions: 95 °C for 5 min, 32 cycles of 95 °C for 30 sec, 60 °C for 30 sec, and 72 °C for 1 min, followed by 72 °C for 10 min.

### Southern blot

Genomic DNA from the transgenic rats was digested with BamHI and EcoRV at 37 °C for 16 hours, and then separated on 0.8% agarose gels prior to Southern analysis. The probe was synthesized using the Prime-a-Gene Labeling kit (Promega, lot:0000182475), which used the Neo cassette as the template, and the probe was labeled with alpha-P32dATP.

### ESC derivation

Blastocysts labeled with *RFP* or *GFP* were seeded on mitomycin-C-treated mouse embryonic fibroblasts (MEFs) and cultured in the previously reported standard “2i” rat ESC medium, which was N2B27 supplemented with “2i” (PD0325901 and CHIR99021) and leukemia inhibitory factor (LIF)^27^. Outgrowths were picked manually and trypsinized into single cells with 0.05% trypsin-EDTA. The cells from each outgrowth (one cell line) were cultured in a new well of the plate in rat ESC medium on feeder cells. The medium was changed daily for each cell line, and the cell lines were passaged every other day.

### AP staining and karyotype analysis

AP staining was performed according to the standard manufacturer’s instructions using the alkaline phosphatase kit. The results were observed under an inverted microscope (DMi-8, Leica, Germany). Karyotype analysis was performed according to standard methods^28^.

### Chimera production

Rat blastocysts were collected from 4.5 dpc F344 strain female rats and injected with 10 to 12 *RFP-* or *GFP*-labeled ESCs. The reconstructed embryos were cultured in mR1ECM (246 mOsM)^29^ in a 37 °C incubator with 5% CO_2_ for 30 min. Images of the reconstructed blastocysts were captured on an inverted microscope (DMi-8, Leica, Germany). The chimeric embryos were transferred to the uteri of pseudo-pregnant female SD rats. The chimeric rats were identified by coat color or expression of the fluorescent proteins.

## Additional Information

**How to cite this article**: Li, T. *et al.* Efficient Production of Fluorescent Transgenic Rats using the *piggyBac* Transposon. *Sci. Rep.* **6**, 33225; doi: 10.1038/srep33225 (2016).

## Supplementary Material

Supplementary Information

## Figures and Tables

**Table 1:** Summary of *GFP* and *RFP* pups generated *via PB* transposition.

Injected *piggyBac* vectors	Rat strain	No. of embryos		No. of pups (%)*	
		Injected embryos	Transferred embryos	Live-born pups	*GFP* or *RFP* pups
*PB-GFP*	SD	75	72	38(52.8)	18(25.0)
	DA	54	45	26(57.8)	12(26.7)
*PB-RFP*	SD	42	39	18(46.1)	8(20.1)
	DA	52	41	16(39.0)	6(14.6)

^*^The percentages of pups that expressed *GFP* or *RFP* were calculated based on the numbers of embryos transferred.
