# Supplementary material for: Efficient Production of Fluorescent Transgenic Rats using the piggyBac Transposon
Source: Sci Rep. 2016 Sep 14;6:33225. doi: 10.1038/srep33225 (PMC5021943; doi:10.1038/srep33225)
Supplement: Supplementary Information [file srep33225-s1.doc]

**Title:**

Efficient Production of Fluorescent Transgenic Rats using the *piggyBac* Transposon

**Authors and affiliations:**

Tianda Li^1*^, Ling Shuai^1,2*^, Junjie Mao^1,3*^, Xuepeng Wang^1^, Mei Wang^1^, Xinxin Zhang^1,4^, Leyun Wang^1,4^, Yanni Li^2^, Wei Li^1^, and Qi Zhou^1#^.

**SUPPLEMENTARY INFORMATION**

A. Emission wavelength of the *GFP* rats; the peak is at approximately 510 nm. B. Emission wavelength of *RFP* rats; the peak is at approximately 600 nm.

A. Analysis of the integration of the *GFP* gene in two individual rats. B. The percentage of *RFP*-positive cells in haploid germ cells was assessed by FACS; the DAPI filter was used to detect the signals from the Hoechst-stained DNA. The upper panel shows the FACS data from the diploid ESCs for comparison; the bottom panel shows the wild type control group. C. Image of RFP-positive oocytes at the germ-vesicle (GV) stage; scale bar = 100 μm.

A. Schematic of the inverse PCR. LTR-void BstYI was employed to digest the genome of the transgenic rats, and T4 DNA ligase was used to form the left LRT (L)- or right LTR (R)-containing cycle, which is the basis of inverse PCR. Following the cycles, two sides of the LTR would join together to form a linear version (L&R). After ligation, two rounds of nested PCR (black arrows) were applied to amplify the target DNA.

B and C. Electrophoretogram of inverse PCR for the *RFP-* and *GFP-*positive rats.

D. *PBase* detection of GFP-positive rats using PCR; each band indicated the random integration of the *PBase* gene.

A. Image of F344 strain rat blastocysts injected with RFP-positive rat ESCs; scale bar = 100 μm.

**Table S1. *RFP*/*GFP*-positive F1 individuals generated from F0 rats mated with wild type (WT) rats**

| Individual F0  pups | Background | Sex | Mated | No. of pups born | No. of RFP/GFP-positive pups (%) |
| --- | --- | --- | --- | --- | --- |
| ＃364* | DA(RFP) | Male | DA(WT) | 13 | 5(38.4) |
| ＃378* | DA(GFP) | Female | DA(WT) | 15 | 4(26.7) |

*: These numbers indicated the F0 adult transgenic rats that carried the *RFP* generated by *PB* transposition.

**Table S2. Summary of the chimeric rats generated from *RFP*-**labeled ESCs

| Donor cell line | Background | Cell passage | Recipient embryo | Reconstructed | Full-term pups (%) | Chimeras (%) |
| --- | --- | --- | --- | --- | --- | --- |
| PBES1-1 | DA(RFP) | P10 | Blastocysts | 54 | 21 (38.9%) | 6(11.1%) |
| PBES1-2 | DA(RFP) | P11 | Blastocysts | 32 | 18 (56.3%) | 4(12.5%) |

*: The percentages of generated pups and chimeras were calculated from the numbers of reconstructed embryos.

**Table S3. Primers used in this study**

| Assay | Primer name | Primer sequence (5' to 3') |
| --- | --- | --- |
| **Inverse PCR** |  |  |
|  | LEFT-L#1 | CCTCGATATACAGACCGATAAAACA |
|  | LEFT-L#2 | CACATGATTATCTTTAACGTACGTCACAAT |
|  | LEFT-R#1 | TCCTCTGAACGCTTCTCGCT |
|  | LEFT-R#2 | CTGCTCTTTGAGCCTGCAGACA |
|  | RIGHT-L#1 | GATGAATCCAGAAAAGCGGCCA |
|  | RIGHT-L#2 | TCCACCATGATATTCGGCAAGCA |
|  | RIGHT-R#1 | GACTGAGATGTCCTAAATGCACAGC |
|  | RIGHT-R#2 | GAGCAATATTTCAAGAATGCATGCGTC |
| **RT- PCR** |  |  |
|  | Oct4-F | GAAGGTGGAACCTAGTCCCGA |
|  | Oct4-R | TGTACCCCAAGGTGATCCTC |
|  | Nanog-F | GCCCTGAGAAGAAAGAAGAG |
|  | Nanog-R | CGTACTGCCCCATACTGGAA |
|  | Rex1-F | TTCTTGCCAGGTTCTGGAAGC |
|  | Rex1-R | TTTCCCACACTCTGCACACAC |
|  | Nestin-F | AGAGAAGCGCTGGAACAGAG |
|  | Nestin-R | AGGTGTCTGCAACCGAGAGT |
|  | Kdr-F | ATACACCTGCACAGCGTACAG |
|  | Kdr-R | TCCCGCATCTCTTTCACTCAC |
|  | Sox17-F | AGGAGAGGTGGTGGCGAGTAG |
|  | Sox17-R | GTTGGGATGGTCCTGCATGTG |
|  | Gapdh-F | ACCACAGTCCATGCCATCAC |
|  | Gapdh-R | TCCACCACCCTGTTGCTGTA |
| **Fluorescent cassettes** |  |  |
|  | RatRFP-F | GGGTAAACTGGGAAAGTGATGT |
|  | RatRFP-R | AATATCACGGGTAGCCAACG |
|  | RatGFP-F | ATGGTGAGCAAGGGCGAG |
|  | RatGFP-R | TTACTTGTACAGCTCGTCCATGCC |
| *PBase* |  |  |
|  | *PBase*-F | TGAGCATGGTGTACGTGTCC |
|  | *PBase*-R | AGCTGTTGATGCAGGCGATA |
